# Antibiotic Activity of Actinobacteria from the Digestive Tract of Millipede *Nedyopus dawydoffiae* (Diplopoda)

**DOI:** 10.3390/antibiotics7040094

**Published:** 2018-10-29

**Authors:** Alla A. Glukhova, Anna A. Karabanova, Andrey V. Yakushev, Irina I. Semenyuk, Yuliya V. Boykova, Natalia D. Malkina, Tatiana A. Efimenko, Tatiana D. Ivankova, Larissa P. Terekhova, Olga V. Efremenkova

**Affiliations:** 1 Gause Institute of New Antibiotics, 119021 Moscow, Russia; alglukhova@yandex.ru (A.A.G.); bird_dodo@mail.ru (A.A.K.); Boykowa.yulya@yandex.ru (Y.V.B.); utvar@blagoslovenie.su (N.D.M.); efimen@inbox.ru (T.A.E.); tiwankova@yandex.ru (T.D.I.); terekhova@list.ru (L.P.T.); 2 Faculty of Soil Science, Lomonosov Moscow State University, 119991 Moscow, Russia; a_yakushev84@mail.ru; 3 A.N. Severtsova Institute of Ecology and Evolution, Russian Academy of Sciences, 119071 Moscow, Russia; free-cat@bk.ru; 4 Joint Russian-Vietnamese Tropical Center, Ho Chi Minh City, Vietnam

**Keywords:** *Streptomyces* spp., *Nedyopus dawydoffiae*, Diplopoda, microbiota, antibiotic activity, drug resistance

## Abstract

Because of the spread of drug resistance, it is necessary to look for new antibiotics that are effective against pathogenic microorganisms. The purpose of this study was to analyse the species composition of actinobacteria isolated from the digestive tract of the millipedes *Nedyopus dawydoffiae* and to determine their antimicrobial properties. Species identification was carried out on the basis of the morphological and culture properties and the sequence of the 16S rRNA gene. Actinobacteria were grown in different liquid media. Antibiotic properties were determined against some Gram-positive and Gram-negative bacteria as well as fungi. Of the 15 isolated strains, 13 have antibiotic activity against Gram-positive bacteria (including methicillin-resistant *Staphylococcus aureus*—MRSA) and fungi, but there was no antibiotic activity against Gram-negative test strains *Escherichia coli* ATCC 25922 and *Pseudomonas aeruginosa* ATCC 27853. It was established that antibiotic-producing actinobacteria belong to eight species of the genus *Streptomyces*. Depending on the nutrient medium, actinobacteria demonstrate different antimicrobial activities. As an example, *S. hydrogenans* shows that even strains selected in one population differ by the range of antimicrobial activity and the level of biosynthesis. Since the antibiotic production is considered as a feature for species competition in the microbiota community, the variability of antibiotic production among different strains of the same species is an adaptive characteristic for the competition in millipedes’ digestive tract community.

## 1. Introduction

The introduction of antibiotics into medical practice was one of the most important achievements of the twentieth century, but pathogenic microorganisms formed antibiotics resistance in response. In connection with this, nowadays antibiotics are becoming increasingly ineffective. According to the World Health Organization (WHO), due to the lack of effective antibiotics against infectious diseases, 700,000 people die in the world every year, and by the expectation by 2050 this indicator will go up one level with the death rate from cancer and cardiovascular diseases [1].

The main producers of known natural antibiotics along with fungi are actinobacteria, mainly representatives of the genus *Streptomyces*; these bacteria and their antibiotic production are well studied [2,3]. Since antibiotics are secondary metabolites, strains of the same species may differ by antibiotics they produce. For discovering new promising compounds it is expedient to investigate strains of actinobacteria—potential producers of antibiotics isolated from poorly studied natural habitats. One of barely studied natural habitat of bacteria is digestive tract of invertebrates, particularly millipedes (Diplopoda). Class Diplopoda contains more than 7500 species, mainly saprophagous animals inhabiting leaf litter and upper soil; millipedes play an important role in biodegradation and soil formation [4,5]. Millipedes have very rich and diverse gut microbiota which helps them to digest material enriched by cellulose, lignin and other plant polymers [4,5,6]. Studies of the microbiota from millipedes digestive tracts showed the presence of various types of microorganisms, including actinobacteria [6,7,8,9,10,11]. Bacteria community also is place-specific for different parts of millipedes’ digestive tract and stays stable while millipedes are starving or change diet [10,11]. Some of actinobacteria in millipedes’ gut community produce antibiotics and enzymes against other microorganisms including species of the same community [12,13,14,15]. Bacterial species appeared as host-specific to different millipede species, so investigating gut microbiota of new millipede species is important for detecting new antibiotics produced by bacteria. 

**Figure 1:**
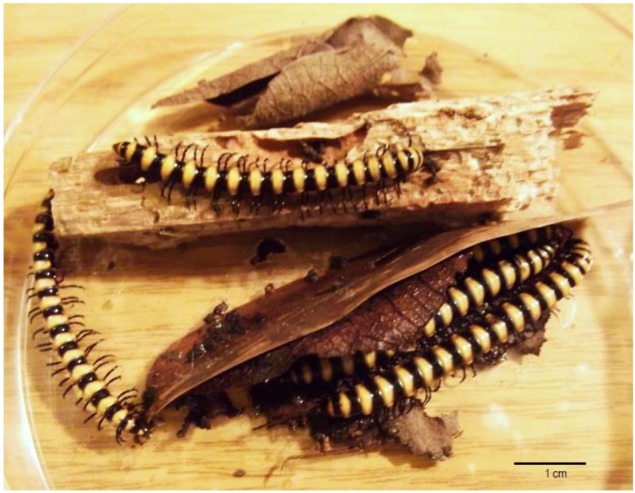
Millipedes *Nedyopus dawydoffiae* (Diplopoda) on rotten wood in a Petri dish.

The objects of this study were actinobacteria from the digestive tract of the millipedes *Nedyopus dawydoffiae* (Diplopoda) collected in Vietnam (Figure 1). *N. dawydoffiae* was chosen as one of the millipede species of the tropical monsoon forest of Southern Vietnam, actively participating in the biodegradation of plant debris, especially tree trunks. We assumed that this microbial model object would be promising as a source of potentially interesting actinobacteria, because these millipedes consume rotten wood, and besides their own microbiota contain many different transitional microorganisms. Competition between microorganisms in particular is expressed in the biosynthesis of antibiotics.

It is known that manufacture of antibiotics is a complex multistage process that takes a long time and includes biological, chemical, preclinical and clinical studies. Of the 30,000–40,000 known natural antibiotics, only about 150 are used in medicine [2,3]. The choice of an object at a biological stage for subsequent chemical study largely determines the efficiency of finding a new natural antibiotic. The biological stage includes the isolation of potential producers of antibiotics into culture, the development of cultivation conditions, the determination of the spectrum of antimicrobial activity, species identification, analysis of previous literature on antibiotics of these species and as a result the selection of the most promising objects for the chemical and subsequent stages of research. The main purpose of this study was analysis of antibiotic properties of millipedes’ actinobacteria. This paper presents the strains selected for further chemical study.

## 2. Results and Discussion

**Figure 2:**
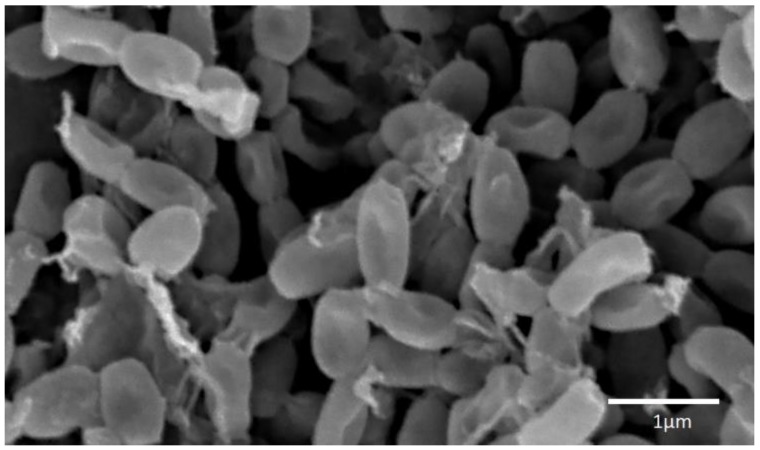
Strain INA 01180 spores. For SEM actinobacterium was cultivated on medium ISP 3 for 12 days.

Fifteen strains of actinobacteria were isolated, 13 of which showed antimicrobial activity during submerged cultivation in one or more nutrient media. On the basis of morphological and cultural characters, when growing on diagnostic media, it was established that these 13 strains belong to eight species of the genus *Streptomyces*. According to the composition of 16S rRNA gene, representatives of five of the eight species of the described actinobacteria, namely *S. fimicarius*, *S. griseoplanus*, *S. pratensis*, *S. setonii* and *S. spororaveus*, correspond to type strains of these species in the GenBank database (Table 1). Strain INA 01180 according to morphological features corresponds to the species *S. globisporus*: the strain forms direct spore chains, the spore shell is smooth (Figure 2), the melanoid pigments are not formed; on media No. 1 Gause, ISP3 and glycerin-nitrate agar forms a mealy pale yellow aerial mycelium, the substrate mycelium is colorless, the soluble pigments are not formed; on medium No. 2 Gause it forms a lean aerial mycelium, substrate mycelium is colorless, soluble pigments are absent [16,17]. Since only 568 bp were read by this method, the sequence of the 16S rRNA gene of this strain coincides with a probability of 99.3% with a large number of different species of streptomycetes. Thus, the species affiliation of strain INA 01180 was determined only on the basis of morphological and cultural features.

**Figure 3:**
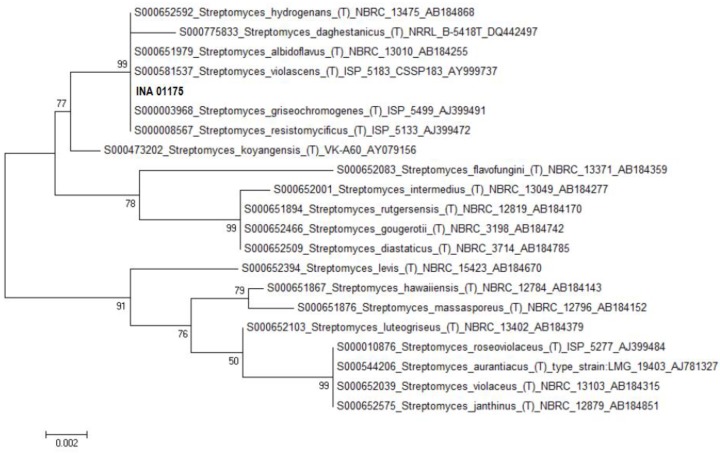
Phylogenetic tree of the strain INA 01175.

**Figure 4:**
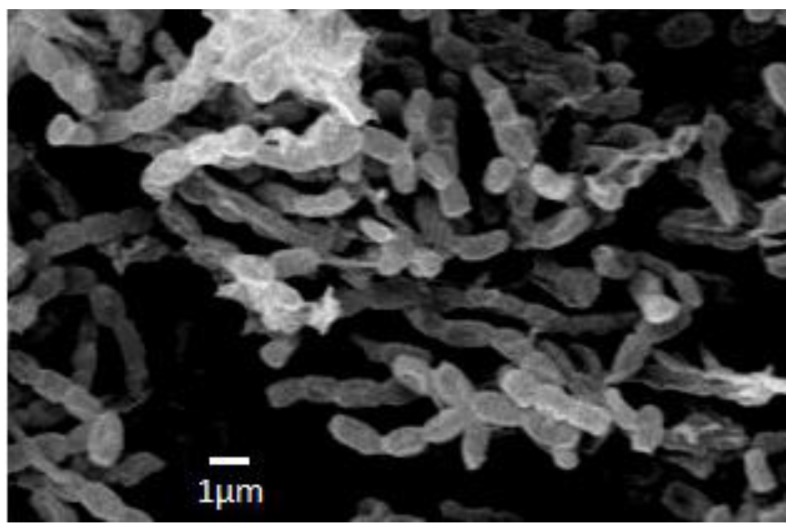
Spore chains of the strain INA 01175. For SEM actinobacterium was cultivated on medium ISP 3 for 12 days.

The strain INA 01175 with the almost completely established sequence of the 16S rRNA gene (1387 bp) shows 100% coincidence with the DNA sequences of type strains of three species of streptomycetes: *S. griseochromogenes*, *S. resistomycificus* and *S. hydrogenans* (Figure 3). Among the listed species, strain INA 01175 is closest to the species *S. hydrogenans*: it forms straight spore chains and the surface of the spores is smooth (Figure 4). When growing on diagnostic media, the pigmentation of the air and substrate mycelium corresponds to the pigmentation of *S. hydrogenans* [16,17]. Among the 15 isolated actinobacteria, four strains belong to this species and they possess these features, but they differed from other previously tested strains of *S. hydrogenans* by the formation of a brown-green pigment on an oat medium (medium ISP 3), which is not typical for *S. hydrogenans*. Thus, the strains INA 01173-01176 and its three analogue strains are closest to the *S*. *hydrogenans*, but it is also possible that they belong to the new species of *Streptomyces* (section Cinereus, series Achromogenes). This question requires additional study.

**Figure 5:**
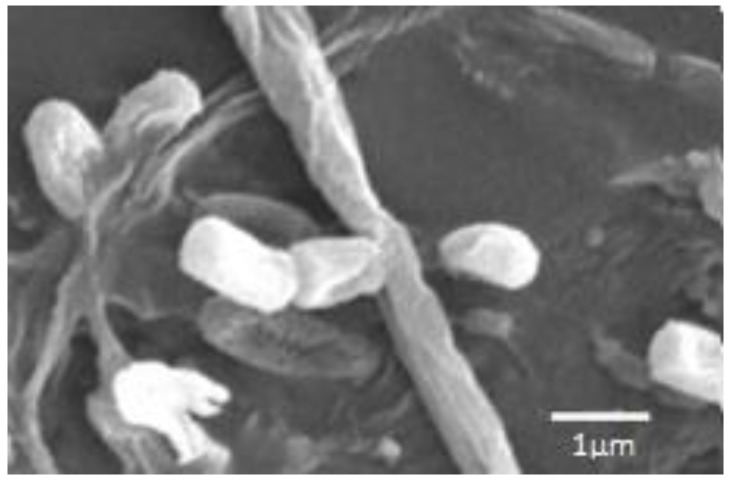
Strain INA 01184 spores. For SEM actinobacterium was cultivated on medium ISP 3 for 12 days.

**Figure 6:**
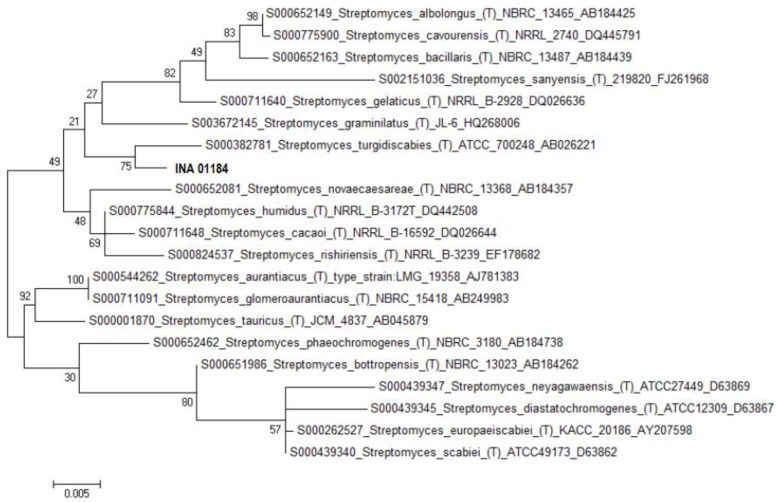
Phylogenetic tree of the strain INA 01184.

According to the sequence of the 16S rRNA gene, the strain INA 01184 corresponds most closely to the type strain of *S. turgidiscabies* ATCC 700248, although the coincidence is only 96.5% (Table 1). For such important morphological features as flexuous spore chains and cylindrical spores with a smooth surface (Figure 5), the strain INA 01184 corresponds to the species *S. turgidiscabies*. Initially, this species was isolated and described in eastern Hokkaido as one of the causative agents of potato scab [18]. Nevertheless, the strain INA 01184, when grown on ISP 3 and No. 1 Gause media, is characterized by a pinkish colour of the soluble pigment and substrate mycelium, as well as the white colour of the spore forming aerial mycelium, although *S. turgidiscabies* is characterized by the grey colour of the spore mass and the absence of soluble pigments released into the medium. A distinctive property of the strain INA 01184 is also the absence of growth at 37 °C. According to morphophysiological characteristics the strain more corresponds to the species *S. acidiscabies*. Both species belong to the group of streptomycetes that cause potato scab (potato scab). Hybridization of the total DNA of type strains of these two species was 20% [19]. According to the GenBank database, type strains of *S. turgidiscabies* SY9113T (=ATCC 700248T = IFO 16080T) and *S. acidiscabies* RL-110 (=ATCC 49003) are not related (Figure 6).

All 13 strains have antimicrobial activity against Gram-positive bacteria and/or fungi (Table 2). There was no antibiotic activity against Gram-negative test strains *Escherichia coli* ATCC 25922 and *Pseudomonas aeruginosa* ATCC 27853. Table 2 is displaying that many actinobacteria, depending on the nutrient medium, form several antibiotics that differ by antimicrobial spectrum. Thus, *S. fimicarius* INA 01179 produces at least two antibiotics—one of which is active against staphylococci when it grows on media B, C, D and F, and the other is active against *Sac. cerevisiae* INA 01129 when it grows on medium E. It is known that *S. fimicarius* forms an antitumor antibiotic, a topoisomerase inhibitor that inhibits the growth of murine leukemia cells P388 [20]. Antibiotics griseorubins, active against Gram-positive and Gram-negative bacteria, mycoplasma, protozoa, leukemia L1210 and Zajdela ascites hepatoma, were also described but there is no information on their antifungal activity [21,22]. Among representatives of *S. globisporus* the producers of antitumor antibiotics lidamycin, landomycins and actinoxanthins, which also possess antibacterial properties, were described. Antifungal activity has not been previously described [23,24,25,26,27,28]. It is possible that strain *S. globisporus* INA 01180 forms one of these antibiotics, as well as an unidentified antifungal antibiotic.

*S. griseoplanus* INA 01177 forms at least three antibiotics which are active against staphylococci, *B. subtilis* ATCC 6633 and *Sac. cerevisiae* INA 01129 (Table 2). It is known that the cultures of this species produce two antitumor antibiotics, alazopeptin and anticapsin, that are also active against trypanosomes as well as Gram-positive bacteria and fungi respectively [29,30,31,32,33]. This species is also known as a producer of the known medical antibiotic erythromycin A, which is characterized by a broad spectrum of antibacterial activity [34].

*S. hydrogenans* INA 01175 when it grows in all media exhibits antifungal activity against *A. niger* INA 00760 and *Sac. cerevisiae* INA 01129. The strain in media C-G forms an antibiotic or antibiotics active against all tests of gram-positive bacteria and/or fungi. In medium B it produces antibiotic exclusively with antifungal activity. Previously, the species *S. hydrogenans* was actively investigated for the purpose of creating a biological product against various fungi, insects and nematodes—pests of agricultural plants [35,36,37]. The structure of one of the antibiotics with antifungal action is 10-(2,2-dimethyl-cyclohexyl)-6,9-dihydroxy-4,9-dimethyl-dec-2-enoic Acid Methyl Ester [38,39]. Also, the strains of this species produce a known antitumor antibiotic actinomycin D, identified by antibiotic activity against bacterial and fungal phytopathogens (*Agrobacterium tumefaciens*, *Pseudomonas syringae*, *Xanthomonas campestris*, *Botrytis allii*, *Fusarium oxysporum* and *Ustilago maydis*) [40].

*S. pratensis* INA 01182 forms at least two antibacterial antibiotics active against *Bacillus subtilis* ATCC 6633 (formed on media A, E) as well as a compound active against both *Bacillus subtilis* ATCC 6633 and *Staphylococcus aureus* INA 00761 (formed on medium F). It is possible that one of them corresponds to the previously described antibacterial antibiotic Methoxyphenyl-Oxime [41].

*S. setonii* INA 01181 shows activity against *Aspergillus niger* INA 00760 as well as Gram-positive bacteria except methicillin-resistant *Staphylococcus aureus* INA 00761 with multiple resistance to antibiotics. Two antibiotics have been previously described for this species: FR109615 (cis-2-aminocyclopentane-1-carboxylic acid) with antifungal activity and A83094A (16-Deethylindanomycin) that is active against Gram-positive bacteria as well as coccidian [42,43]. 

*S. spororaveus* INA 01183, when grown in all media, has antifungal activity previously described for phythopatogenic fungi [44]. In addition, this strain shows antibacterial activity against gram-positive bacteria, including resistant *Staphylococcus aureus*.

*S. turgidiscabies* INA 01184 has only antifungal activity, which is manifested in relation to *A. niger* INA 00760 and *Sac. cerevisiae* INA 01129 when it grows in medium C or only with respect to *A. niger* INA 00760 when it grows in media A, E, G, which suggests the ability of this strain to form two antifungal agents. Perhaps one of them corresponds to the previously described antibiotic antimycin A [45].

**Figure 7:**
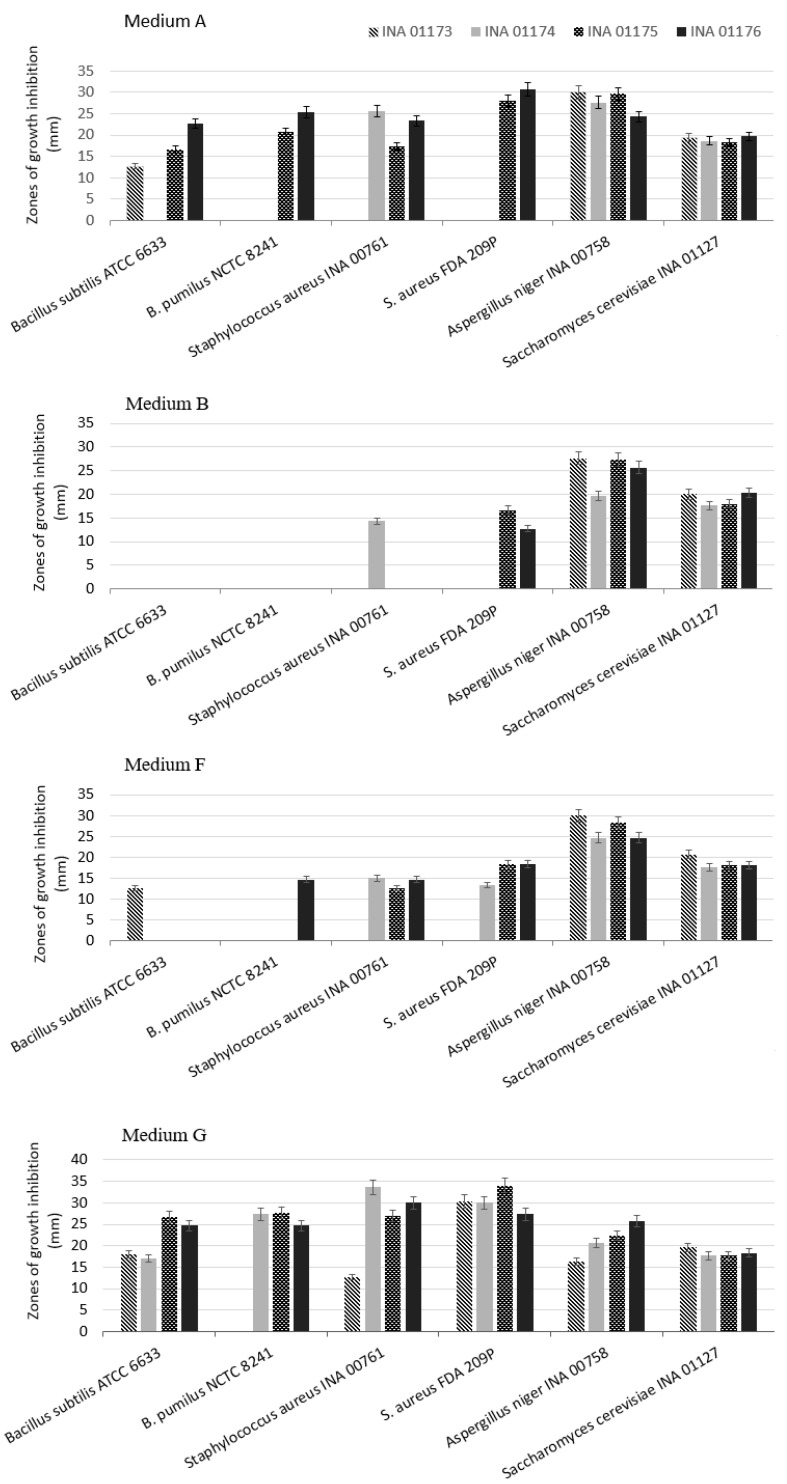
Antimicrobial activity of four bacterial strains *S. hydrogenans* which were cultivated on different media. The mean values are shown; the ±SD is also shown (*n* = 3).

Since strains of the same species may differ by the produced antibiotic, we compared the antimicrobial activity of the four strains *S. hydrogenans* isolated from millipede. It has been established that even strains isolated from one population differ in the spectrum of antimicrobial activity displayed and in the level of biosynthesis. Antifungal activity is characteristic of all four strains at a high level, when they grow in all seven media, but antibacterial activity varies. Figure 7 shows antibiotic activity in growth in four media out of seven, in which the differences are more pronounced. The medium G is most favourable for all four strains *S. hydrogenans* for the manifestation of antibiotic activity. When grown in medium B and in most cases in medium F, antibacterial activity is absent in most strains. It follows from Figure 7 that these strains form various antibacterial compounds. Unlike the three other strains of *S. hydrogenans*, strain INA 01173 does not have activity against *Bacillus pumilus* NCTC 8241 in any of media. Such variability possibly is adaptive characteristic of bacteria population for the species competition in the microbial community of the millipedes’ digestive tract.

## 3. Materials and Methods

**The objects of research** were actinobacteria isolated from the digestive tract of millipedes *Nedyopus dawydoffiae* Attems, 1953 (Diplopoda, Polydesmida). The millipedes were collected by hands in tropical monsoon lowland forest in the Cat Tien National Park (Southern Vietnam, 11°25′ N, 107°25′ E, about 120 m a.s.l.); for more details about park vegetation and climate see the follow article [46]. *N. dawydoffiae* usually appears on decaying logs and consumes rotten wood, moss and leaf litter. In the laboratory millipedes were kept in microcosms with room temperature and humidity 250% (water:substratum = 2.5:1) on rotten wood as natural substratum collected from the same habitat. For irrigation the distilled water was used. For obtaining the gut content 5 millipedes were killed by ethyl acetate and dissected in the sterile conditions. The contents of the digestive tracts of all five individuals were combined, suspended in distilled water, passed through a cotton filter, and seeded on agar medium No. 1 Gause. After 10–12 days of incubation at 37 °C, the colonies of actinobacteria were transferred into tubes on the same medium and agar medium No. 2 Gause. 

**Test-strains.** The following microorganisms were used as test strains for determination of antimicrobial activity: *Bacillus subtilis* ATCC 6633, *B. pumilus* NCTC 8241, *Staphylococcus aureus* FDA 209P (methicillin-sensitive *Staphylococcus aureus*—MSSA), *S. aureus* INA 00761 (methicillin-resistant *Staphylococcus aureus*—MRSA), *Escherichia coli* ATCC 25922, *Pseudomonas aeruginosa* ATCC 27853, *Aspergillus niger* INA 00760, *Saccharomyces cerevisiae* INA 01129.

**Nutrient media.** To maintain the viability of actinobacteria, the Gause agar medium No. 2 was used (%): glucose—1, peptone—0.5, tryptone—0.3, NaCl—0.5, agar—2, tap water, pH 7.2–7.4. For the description of cultural and morphological characters the next diagnostic media were used: medium No. 1 Gause, glycerin-nitrate agar, media ISP3, ISP4 and ISP5 [16,17].

Submerged cultivation of actinobacteria was carried out in eight liquid nutrient media developed for actinobacteria—producers of antibiotics at Gause Institute (Table 3).

**Cultivation conditions:** Actinobacteria were incubated at 28 °C on the above agar media for 10–12 day. Bacterial test strains were grown on medium No. 2 Gause at 37 °C during 24 h. Fungal test strains *A. niger* INA 00760 and *Sac. cerevisiae* INA 01129 were grown at 28 °C on the same medium for 48 and 24 h respectively.∙

Submerged cultivation of actinobacteria was carried out in two stages. To do this, 750 mL Erlenmeyer flasks with 150 mL of medium in both stages were used. The flasks were placed on rotary shakers with a rotation speed of 200 rpm. In order to obtain a submerged inoculum at the first stage, the medium H was used, which was inoculated with a piece of agar medium with an area of about 1 cm^2^ with surface growth of actinobacteria. After 4 days of cultivation for the implementation of the second stage of cultivation, 5 mL of the obtained inoculum was injected into flasks with seven other media A-G and incubated for seven days.

**Scanning electron microscopy (SEM):** Investigations of actinobacterial spores were performed on a scanning electron microscope JEOL-6060A (JEOL, Tokyo, Japan) with a wolfram cathode. Before investigation platinum was sprayed on the spore samples in JFC-1600 unit (JEOL, Tokyo, Japan).

**Determination of antibiotic activity:** The antibiotic activity of the actinobacterial cultural liquid was determined by diffusion in agar. For this purpose, 0.1 mL of cultural liquid was added to the holes in agar medium inoculated with test strains. After incubation during 20–24 h diameters of the growth-inhibitory zones of the test strains were measured as indicator of antibiotic activity.

**Species identification of actinobacteria:** For species identification, such features as the structure of sporophores, the spore surface, the pigmentation of the air mycelium as well as substrate mycelium, and the pigment released into the medium were taken into account. The sequence of the 16S rRNA gene was also taken into account. For DNA isolation used three-day biomass obtained in liquid medium H. Isolation of genomic DNA from the biomass of actinobacteria was carried out using the PowerSoil DNA Kit (MO BIO, Carlsbad, CA, USA). PCR of the 16S rRNA gene was performed using a set of PCR Master Mix reagents (contains *Taq* DNA polymerase; Thermo Scientific, Foster City, CA, USA) with universal bacterial primers 27f (aga gtt tga tcc tgg ctcag) and 1492r (tac ggy tac ctt gtt acg act t). PCR was performed on a Thermal Cycler 2720 device (Applied Biosystems, USA) according to the program: (1) 94 °C for 5 min, (2) 30 cycles with alternating temperature intervals of 94 °C for 1 min, 51 °C for 1 min, 72 °C for 2 min, (3) 72 °C for 7 min. Analysis of PCR products was performed by electrophoresis in a 1% agarose gel (using TBE Tris-borate buffer) at an electric field strength of 7.6 V/cm. Purification of PCR products was carried out by reprecipitation of DNA under mild conditions using 0.125 M ammonium acetate in 70% ethanol. The nucleotide sequences were determined by the Sanger method on automatic sequencer Genetic Analyzer 3500 (Applied Biosystems, Beverly, MA, USA) using universal bacterial primers 27f, 341f (cct acg gga ggc agc ag), 519r (gta tta ccg cgg ctg ctg), 785f (ggm tta gat acc tgg tag tcc), 907r (ccg tca att cct ttg agt tt), 1100r (ggg ttg cgc tcg ttg), 1114f (gca acg agc gca acc c), 1492r. The Mega 7 program was used to assemble the nucleotide sequences. The sequences obtained were compared with the nucleotide sequences of the 16S rRNA gene of the actinobacterial type strains from the GenBank databases (blast.ncbi.nlm.nih.gov/Blast.cgi) and the Ribosomal Database Project (RDP rdp.cme. msu.edu/). 

## 4. Conclusions

Actinobacteria of microbiota of the digestive tract of *Nedyopus dawydoffiae* in overwhelming majority are producers of antibiotic substances against fungi and Gram-positive bacteria, including methicillin-resistant strain *Staphylococcus aureus* (MRSA). The function of antibiotics for actinobacterial producers can be different, but in multi-species biocenosis, such as the microbiota of the digestive tract of the millipedes they can act as chemical weapons in the interspecific struggle. Presumably, the more complex the microbiota, the more diverse the spectrum of antibiotic activity of its microorganisms, in addition, microorganisms of the same species may differ by antibiotic spectrum. In this regard, we consider it expedient to search for producers of antibiotics among representatives of gut microbiota of millipedes.

## Figures and Tables

**Table 1:** Species affiliation of streptomycete strains on the basis of 16S rRNA gene analysis.

Species, Strains	DNA (bp)	Alignment (%) *	ID of the Deposited Sequences (GenBank)
*S. fimicarius* INA 01179	1436	99.4	MH635263
*S. griseoplanus* INA 01177	1485	99.2	MH635265
*S. hydrogenans* INA 01175 **	1387	100	-
*S. hydrogenans* INA 01173 **	1116	100	-
*S.* sp. INA 01174 ***	551	100	-
*S.* sp. INA 01176 ***	569	100	-
*S. pratensis* INA 01182	1426	98.4	MH635266
*S. setonii* INA 01181	1468	100	MH635267
*S. spororaveus* INA 01183	1451	100	MH635268
*S. turgidiscabies* INA 01184 **	1309	96.5	-
*Streptomyces* sp. INA 01180 ****	568	99.3	MH635264

* The percentage of alignment with the gene sequences of the type strains 16S rRNA from the GenBank database. ** By alignment of the sequences of the 16S rRNA gene, these strains are the closest to the indicated species according to the GenBank data, but they do not completely correspond to them by morpho-cultural features. *** By their morphological and cultural characteristics, these strains identical to strains *S. hydrogenans* INA 01174 and INA 01176. **** The strain INA 01180 according to morphological features corresponds to the species *S. globisporus*.

**Table 2:** Spectra of antimicrobial activity of actinobacteria when they grow in different liquid nutrient media.

Species, Strains	Media	*Bacillus subtilis* ATCC 6633	*Bacillus pumilus* NCTC 8241	*Staphylococcus aureus* INA 00761 (MRSA)	*Staphylococcus aureus* FDA 209P (MSSA)	*Aspergillus niger* INA 00760	*Saccharomyces cerevisiae* INA 01129
*S. fimicarius* INA 01179	A, G	−	−	−	−	−	−
	B, C, D, F	−	−	++	+++	−	−
	E	−	−	−	−	−	++
*S. globisporus* INA 01180	A, B, G	−	−	−	−	−	−
	C, D, F	−	−	+++	+++	−	−
*S. griseoplanus* INA 01177	A	−	−	−	−	−	−
	B, F	−	−	+++	++++	−	−
	C	++	−	+++	++	−	−
	D	−	−	++	++	−	−
	E	−	−	++	++	−	++
	G	+++	−	−	−	−	−
*S. hydrogenans* INA 01175	A	+	−	−	+++	++++	+++
	B	−	−	−	−	++++	+++
	C	+	+	++	+++	++++	+++
	D, E, F, G	++++	++++	++++	++++	++++	+++
*S. pratensis* INA01182	A, E	++	−	−	−	−	−
	B, D, G	−	−	−	−	−	−
	F	++	−	+++	−	−	−
*S. setonii* INA 01181	A	++	++	−	+	+++	nd
	B	−	++	−	++	−	nd
	C	+	+++	−	−	+++	nd
	D	++	−	−	−	+++	nd
	E	++++	++++	−	+++	++++	nd
	F	++	++++	−	++	−	nd
	G	−	−	−	−	−	−
*S. spororaveus* INA 01183	A, F, G	++	++	−	−	+++	++
	B, D	−	−	−	−	++	−
	C, E	+++	+++	+++	++	++++	++
*S. turgidiscabies* INA 01184	A, E, G	−	−	−	−	++	−
	B, D, F	−	−	−	−	−	−
	C	−	−	−	−	+++	++

Zones (mm) of test strains suppression as an indicator of antibiotic activity intensity: «−»—no activity, «+»—≤ 10, «++»—11–15, «+++»—16–20, «++++»—> 20, «nd»—no data.

**Table 3:** Nutrient media for the submerged cultivation of actinobacteria, developed at the Gause Institute.

Name	Organic Components (%)	Salts (%)	Water	pH
A (2663)	glycerin—3, soy flour—1.5,	NaCl—0.3, chalk—0.3,	tap	7.0
B (A4)	glucose—1, soy flour—1	NaCl—0.5, chalk—0.25	tap	6.8
C (Suc)	sucrose—2, soy flour—1	NaCl—0.3, chalk—0.3	tap	6.8–7.0
D (5339)	glycerin—2, soy flour—0.5	(NH_4_)_2_SO_4_—0.15, NaCl—0.3, chalk—0.3	tap	6.8
E (6613)	starch—2, corn extract—0.3	KNO_3_—0.4, NaCl—0.5, chalk—0.5	tap	7.0–7.2
F (330)	sucrose—2.1, starch—0.85, pea flour—1.5	NaCl—0.5, NaNO_3_—0.5, chalk—0.5	tap	7.0
G (Am)	sucrose—4, yeast extract—0.25	K_2_HPO_4_—0.1, Na_2_SO_4_—0.1, NaCl—0.1, (NH_4_)_2_SO_4_—0.2, FeSO_4_ 7H_2_O—0.0001, MnCl_2_ 4H_2_O—0.0001, NaI—0.00005, chalk—0.2	distilled	6.5–6.7
H (STR)	glucose—1, peptone—0.5, tryptone—0.3	NaCl—0.5	tap	7.2–7.4

## References

[1] O’Neill J. (2016). The Review on Antimicrobial Resistance. Tackling Drug-Resistant Infections Globally: Final Report and Recommendations. http://amr-review.org/sites/default/files/160518_Final%20paper_with%20cover.pdf.

[2] Bérdy J. (2005). Bioactive microbial metabolites. J. Antibiot..

[3] Bérdy J. (2012). Thoughts and facts about antibiotics: Where we are now and where we are heading. J. Antibiot..

[4] Hopkin S.P., Read H.J. (1992). The Biology of Millipedes.

[5] Zhang Z.-Q. (2013). Animal biodiversity: An outline of higher-level classification and survey of taxonomic richness (Addenda 2013). Zootaxa.

[6] Dhivya A., Alagesan P. (2017). Millipedes as Host for Microbes (Review). Int. J. Microbial. Res..

[7] Byzov B.A., Zenova G.M., Babkina N.I., Dobrovolskaya T.G., Tretjakova E.B., Zvyagintsev D.G. (1993). Actinomycetes in the food, gut and faeces of soil millipede, Pachyiulus flavipes C.L. Koch. Microbiology.

[8] Byzov B.A., König H., Varma A. (2006). Intestinal Microbiota of Millipedes. Intestinal Microorganisms of Termites and Other Invertebrates.

[9] Chu T.L., Szabo I.M., Szabo I. (1987). Nocardioform gut actinomycetes of *Glomeris hexasticha* Brandt (Diplopoda). Biol. Fert. Soils.

[10] Jager K., Marialigeti K., Hauck M., Barabas G. (1983). *Promicromonospora enterophila* sp. nov., a New Species of Monospore Actinomycetes. IJSB.

[11] Knapp B.A., Seeber J., Rief A., Meyer E., Insam H. (2010). Bacterial community composition of the gut microbiota of *Cylindroiulus fulviceps* (diplopoda) as revealed by molecular fingerprinting and cloning. Folia Microbiol..

[12] Byzov B.A., Thanh V.N., Bab’eva I.P., Tretyakova E.B., Dyvak I.A., Rabinovich Y.M. (1998). Killing and hydrolytic activities of the gut fluid of the millipede *Pachyiulus flavipes* C.L. Koch on yeast cells. Soil Biol. Biochem..

[13] Jarosz J., Kania G. (2000). The question of whether gut microflora of the millipede *Ommatoiulus sabulosus* could function as a threshold to food infections. Pedobiologia.

[14] Marialigeti K., Contreras E., Barabas G., Heydrich M., Szabo I.M. (1985). True intestinal actinomycetes of Millipedes (Diplopoda). J. Invertebr. Pathol..

[15] Nguyen Duc T.L., Byzov B.A., Zenova G.M., Zveagintsev D.G. (1996). Antagonistic properties of actinomycetes associated with intestinal tract of soil invertebrates. Vestnik Mosk. Un-ta.

[16] Gause G.F., Preobrazhenskaya T.P., Sveshnikova M.A., Terekhova L.P., Maksimova T.S. (1983). The Guide for Identification of Actinomycetes.

[17] Shirling E.B., Gottlieb D. (1966). Methods for Characterization of Streptomyces Species. IJSB.

[18] Miyajima K., Tanaka F., Takeuchi T., Kuninaga S. (1998). *Streptomyces turgidiscabies* sp. nov.. IJSEM.

[19] Lambert D.H., Loria R. (1989). *Streptomyces acidiscabies* sp. nov.. IJSEM.

[20] Oka H., Yoshinari T., Murai T., Kawamura K., Satoh F., Funaishi K., Okura A., Suda H., Okanishi M., Shizuri Y. (1991). A new topoisomerase-II inhibitor, BE-10988, produced by a streptomycete. I. Taxonomy, fermentation, isolation and characterization. J. Antibiot..

[21] Dornberger K., Berger U., Knöll H. (1980). Griseorubins, a new family of antibiotics with antimicrobial and antitumor activity. I. Taxonomy of the producing strain, fermentation, isolation and chemical characterization. J. Antibiot..

[22] Dornberger K., Berger U., Gutsche W., Jungstand W., Wohlrabe K., Härtl A., Knöll H. (1980). Griseorubins, a new family of antibiotics with antimicrobial and antitumor activity. II. Biological properties and antitumor activity of the antibiotic complex griseorubin. J. Antibiot..

[23] Li X., Lei X., Zhang C., Jiang Z., Shi Y., Wang S., Wang L., Hong B. (2016). Complete genome sequence of *Streptomyces globisporus* C-1027, the producer of an enediyne antibiotic lidamycin. J. Biotechnol..

[24] Shao R., Zhen Y. (2008). Enediyne Anticancer Antibiotic Lidamycin: Chemistry, Biology and Pharmacology. Anti-Cancer Agents Med. Chem..

[25] Gromyko O., Rebets Y., Ostash B., Luzhetskyy A., Fukuhara M., Bechthold A., Nakamura T., Fedorenko V. (2004). Generation of *Streptomyces globisporus* SMY622 Strain with Increased Landomycin E Production and It’s Initial Characterization. J. Antibiot..

[26] Krohk K., Rohr J. (1997). Angucyclines: Total syntheses, new structures and biosynthetic studies of an emerging new class of antibiotics. Top. Curr. Chem..

[27] Khokhlov A.S., Cherches B.Z., Reshetov P.D., Smirnova G.M., Sorokina I.B., Prokoptzeva T.A., Koloditskaya T.A., Smirnov W., Navashin S.M., Fomina I.P. (1969). Physico-chemical and biological studies on actinoxanthin, an antibiotic from *Actinomyces globisporus* 1131. J. Antibiot..

[28] Khokhlov A.S., Reshetov P.D., Chupova L.A., Cherches B.Z., Zhigis L.S., Stoyachenko I.A. (1976). Chemical studies on actinoxanthin. J. Antibiot..

[29] Patterson E.L., Johnson B.L., DeVoe S.E., Bohonos N. (1965). Structure of the antitumor antibiotic alazopeptin. Antimicrob. Agents Chemother..

[30] Ishiyama A., Otoguro K., Namatame M., Nishihara A., Furusawa T., Masuma R., Shiomi K., Takahashi Y., Ichimura M., Yamada H. (2008). In Vitro and in Vivo Antitrypanosomal Activity of Two Microbial Metabolites, KS-505a and Alazopeptin. J. Antibiot..

[31] Hata T., Umezawa I., Iwai Y., Katagiri M., Awaya J., Komiyama K., Oiwa R., Atsumi K. (1973). Studies on the antitumor activity of an alazopeptin isolated from a new strain of *Streptomyces*. J. Antibiot..

[32] Neuss N., Molloy B.B., Shah R., DeLaHiguera N. (1970). The structure of anticapsin, a new biologically active metabolite of *Streptomyces griseoplanus*. Biochem. J..

[33] Boeck L.D., Christy K.L., Shah R. (1971). Production of anticapsin by *Streptomyces griseoplanus*. Appl. Microbiol..

[34] Thompson R.M., Strong F.M. (1971). Identification of erythromycin A in cultures of *Streptomyces griseoplanus*. Biochem. Biophys. Res. Commun..

[35] Kaur T., Manhas R.K. (2014). Antifungal, insecticidal, and plant growth promoting potential of *Streptomyces hydrogenans* DH16. J. Basic Microbiol..

[36] Kaur T., Vasudev A., Sohal S.K., Manhas R.K. (2014). Insecticidal and growth inhibitory potential of *Streptomyces hydrogenans* DH16 on major pest of India, *Spodoptera litura* (Fab.) (Lepidoptera: Noctuidae). BMC Microbiol..

[37] Manhas R.K., Talwinder K. (2016). Biocontrol Potential of *Streptomyces hydrogenans* Strain DH16 toward *Alternaria brassicicola* to Control Damping Off and Black Leaf Spot of *Raphanus sativus*. Front. Plant. Sci..

[38] Kaur T., Kaur A., Sharma V., Manhas R.K. (2016). Purification and Characterization of a New Antifungal Compound 10-(2,2-dimethyl-cyclohexyl)-6,9-dihydroxy-4,9-dimethyl-dec-2-enoic Acid Methyl Ester from *Streptomyces hydrogenans* Strain DH16. Front. Microbiol..

[39] Kaur T., Jasrotia S., Manhas K. (2016). Evaluation of in vitro and in vivo nematicidal potential of a multifunctional streptomycete, *Streptomyces hydrogenans* strain DH16 against *Meloidogyne incognita*. Microbiol. Res..

[40] Kulkarni M., Gorthi S., Chattopadhyay P., Banerjee G. (2017). Production, characterization and optimization of actinomycin D from *Streptomyces hydrogenans* IB310, an antagonistic bacterium against phytopathogens. Biocatal. Agricult. Biotechnol..

[41] Barghouthi S.A., Ayyad I., Ayesh M., Abu-Lafi S. (2017). Isolation, Identification, and Characterization of the Novel Antibacterial Agent Methoxyphenyl-Oxime from *Streptomyces pratensis* QUBC97 Isolate. J. Antibiot. Res..

[42] Iwamoto I., Tsujii E., Ezaki M., Fujie A., Hashimoto S., Okuhara M., Kohsaka M., Imanaka H., Kawabata K., Inamoto Y. (1990). FR109615, a new antifungal antibiotic from *Streptomyces setonii*. Taxonomy, fermentation, isolation, physico-chemical properties and biological activity. J. Antibiot..

[43] Larsen S.H., Boeck L.D., Mertz F.P., Paschal J.W., Occolowitz J.L. (1988). 16-Deethylindanomycin (A83094A), a novel pyrrole-ether antibiotic produced by a strain of *Streptomyces setonii*. Taxonomy, fermentation, isolation and characterization. J. Antibiot..

[44] Al-Askar A.A., Abdul Khair W.M., Rashad Y.M. (2011). In vitro antifungal activity of *Streptomyces spororaveus* RDS28 against some phytopathogenic fungi. Afr. J. Agric. Res..

[45] Kotiaho M., Aittamaa M., Andersson M.A., Mikkola R., Valkonen J.P.T., Salkinoja-Salonen M. (2008). Antimycin A-producing nonphytopathogenic *Streptomyces turgidiscabies* from potato. J. Appl. Microbiol..

[46] Blanc L., Maury-Lechon G., Pascal J.-P. (2000). Structure, floristic composition and natural regeneration in the forests of Cat Tien National Park, Vietnam: An analysis of the successional trends. J. Biogeogr..

